# Wild-Grown Romanian *Helleborus purpurascens* Approach to Novel Chitosan Phyto-Nanocarriers—Metabolite Profile and Antioxidant Properties

**DOI:** 10.3390/plants12193479

**Published:** 2023-10-04

**Authors:** Adina-Elena Segneanu, Gabriela Vlase, Titus Vlase, Crina Andreea Sicoe, Maria Viorica Ciocalteu, Dumitru Daniel Herea, Ovidiu-Florin Ghirlea, Ioan Grozescu, Valentin Nanescu

**Affiliations:** 1 Institute for Advanced Environmental Research-West, University of Timisoara (ICAM-WUT), Oituz Nr. 4, 300086 Timisoara, Romania; gabriela.vlase@e-uvt.ro (G.V.); titus.vlase@e-uvt.ro (T.V.); 2 Research Centre for Thermal Analysis Environmental Problems, West University of Timisoara, Pestalozzi St. 16, 300115 Timisoara, Romania; 3 Faculty of Chemistry, Biology, Geography, West University of Timisoara, Pestalozzi St. 16, 300115 Timisoara, Romania; crinandreea89@gmail.com; 4 Faculty of Pharmacy, University of Medicine and Pharmacy Craiova, St. Petru Rareș 2, 200349 Craiova, Romania; maria.ciocilteu@umfcv.ro (M.V.C.); valentin.nanescu@gmail.com (V.N.); 5 National Institute of Research and Development for Technical Physics, 47 Mangeron Blvd., 700050 Iasi, Romania; dherea@phys-iasi.ro; 6 Faculty of Medicine, “Victor Babes” University of Medicine and Pharmacy, square Eftimie Murgu No. 2, 300041 Timisoara, Romania; ovidiu.ghirlea@gmail.com; 7 CAICON Department, University Politehnica Timisoara, 300006 Timisoara, Romania; ioangrozescu@gmail.com

**Keywords:** chitosan, nanoencapsulation, carriers, hellebore, antioxidant activity, phytoconstituents, silver nanoparticles

## Abstract

The current nanomedicinal approach combines medicinal plants and nanotechnology to create new scaffolds with enhanced bioavailability, biodistribution and controlled release. In an innovative approach to herb encapsulation in nanosized chitosan matrices, wild-grown Romanian *Helleborus purpurascens* was used to prepare two new chitosan nanocarriers. The first carrier preparation involved the nanoencapsulation of hellebore in chitosan. The second carrier emerged from two distinct stages: hellebore-AgNPs phyto-carrier system succeeded by nanoencapsulation in chitosan. The morphostructural characteristics and thermal behavior of these newly prepared nanocarriers were examined using FT-IR, XRD, DLS, SEM, EDS and thermogravimetric analyses. In addition, the encapsulation yield, encapsulation efficiency and encapsulation contents were investigated. The antioxidant activity was estimated using four in vitro, noncompetitive methods: total phenolic assay; 2,2-diphenyl-1-picrylhydrazyl (DPPH) radical scavenging assay; phosphomolybdate (i.e., total antioxidant capacity); and iron(III)-phenanthroline antioxidant assay. Moreover, this study reports the first low-molecular-weight metabolite profile of wild-grown Romanian *Helleborus purpurascens Waldst. & Kit.* A total of one hundred and five secondary metabolites were identified in the mass spectra (MS)-positive mode from fourteen secondary metabolite categories (alkaloids, butenolides, bufadienolides, phytoecdysteroids, amino acids and peptides, terpenoids, fatty acids, flavonoids, phenolic acids, sterols, glycosides, carbohydrates, nucleosides and miscellaneous). The collective results suggest the potential application is a promising new antioxidant vehicle candidate in tumor therapeutic strategy.

## 1. Introduction

*Helleborus purpurascens Waldst. & Kit*. (*Helleborus purpurascens L*.) from the Ranunculaceae family has been used as a medicinal plant in Romanian and European ethnomedicine for centuries [1,2]. One of the first mentions of the therapeutic effect of this plant occurs in *Diseases of Women* by Hippocrates. Also, the use of the plant in Dacian medicine is reported in *De Materia Medica* [2]. In traditional European medicine, hellebore is used for its antimicrobial, anti-inflammatory, antitumor, cardiotonic, analgesic, antiseizure, laxative and other effects [2,3,4]. Modern Romanian studies from the end of the last century have demonstrated the therapeutic potential of the plant in the treatment of severe forms of rheumatism [1,2,5,6,7]. Recent research has confirmed their unique antitumor and immunomodulating activity [8,9,10,11]. Hellebore’s outstanding biological activity is the outcome of the combined and synergistic action of its multiple bioactive compounds (bufadienolides, butenolides, phytoecdysteroids, terpenoids, alkaloids, sterols, flavonoids, fatty acids, peptides, lactones, phenolic acids, carbohydrates and others) [1,2].

Synthetic drugs are the primary option in the therapeutic strategy for severe ailments. In addition, factors such as pollution, self-medication or others can induce drug resistance and, thus, treatment failure [12]. Conversely, ethnomedicine had an essential role in the development of humanity and is currently the first choice as a treatment strategy for various diseases in over 80% of all countries, according to the WHO’s estimation [13]. In this context, urgent alternative, efficient strategies are required. To this end, intensive efforts have been made to develop new and performant plant-based drug delivery systems. Consequently, substantially modern pharmaceuticals are based on natural products despite the numerous technical barriers related to bioactive compounds, namely, total synthesis and optimization [14,15]. Furthermore, many phytoconstituents with exceptional biological activity display limited in vivo bioavailability and reduced membrane transport [14,16,17]. As a result, numerous studies have focused on new drug delivery systems with designs that encapsulate secondary metabolites in different biomolecules [14,16,17,18].

Alternatively, metal nanoparticles, particularly magnetic or precious metals, have been exploited in numerous biomedical applications because of their remarkable physicochemical and biological characteristics [19,20,21,22]. Among these, silver nanoparticles show huge biopotential (high stability and flexibility, biocompatibility, ability to cross the blood–brain barrier (BBB)) for detection, diagnosis and treatment (drug delivery, antibacterial, antiviral, antifungal, antitumor and antioxidant agents) [23,24,25]. Nonetheless, the main drawback of AgNPs is the genotoxicity and cytotoxicity mechanisms of AgNPs that occur in different tissues or organs under certain conditions (concentration, contact time, dimensions, etc.). The different latest approaches based on AgNP coating/encapsulation or functionalization have overcome these shortcomings. Among the biomaterials used for a targeted drug delivery design, chitosan stands out for its performance and versatility. Chitosan is an incredibly versatile, low-cost substance with numerous applications in diverse fields, such as agriculture, biotechnology, cosmetics, food preservation, medicine, nanotechnology, textiles and wastewater treatment. It is a natural biopolymer composed of various units (2-acetamido-2-deoxy-D-glucopyranose and 2-amino-2-deoxy-D-glucopyranose), and is the next plentifullest natural polymer after cellulose [26,27]. This biopolymer is produced using deacetylating chitin in an alkaline environment. Chitosan is the most suitable material for biomedical applications because of its outstanding features: biocompatibility, nontoxicity, mucoadhesive properties and antibacterial activity [26,27,28]. Moreover, chitosan nanoparticles demonstrate significant potential for cancer and osteoarthritis by providing target specificity, drug stability and efficiency [26,27,28].

Great effort has been devoted to the study of antioxidants’ role in the prevention of cancer and its cure [29,30,31,32]. However, despite their critical potential in tumor therapy, their effectiveness is still a controversial issue in the medical field due to several limitations, namely, low bioavailability and transmembrane permeability, inadequate doses, uneven distribution, among others, which can decrease their beneficial outcomes [31]. Therefore, a novel approach is necessary to ensure the successful delivery of antioxidants and maximize their therapeutic effects. Furthermore, the activity of plant biology is a complex process that relies on the synergistic action of various phytoconstituents in different proportions [32,33,34]. Factors such as climate, soil composition and the plant’s harvesting stage can significantly impact the antioxidant activity of these phytoconstituents. The antioxidant activities of phytoconstituents varies with certain factors: ecological parameters (sunlight, temperature, aridity, humidity, soil composition and pH), harvest growth stage and others [35].

Antioxidant agents are usually classified based on their physical–chemical properties, source and mechanism of action. Additionally, it is worth noting that various criteria, such as metabolism pathway, bioactive phytocompound chemical structure, amount, bioavailability and specific rate constant could significantly affect the effectiveness of an antioxidant agent [35,36,37,38,39]. In conclusion, to gain a comprehensive understanding of phytoconstituents’ biologic activity, it is crucial to simultaneously examine the factors encompassing the origin and composition of the phytoconstituents with the techniques utilized to assess their antioxidant properties.

An herb or plant extract loaded into biopolymer nanoparticles ensures the controlled intake of highly active phytoconstituents, cancels the effects of overdose-induced tissue damage and increases the therapeutic efficiency compared to individual components because of the complementary action of the plant and the biopolymer [26,27]. Moreover, nanocarrier systems present multiple advantages compared to other drug delivery systems: improved efficiency through the complementary action between encapsulated biomolecules and chitosan nanoparticles; higher stability; fast, selective, targeted and sustained release; and excellent biocompatibility. In this sense, the research on developing successful phyto-carriers based on nanoparticles reflects a substantial improvement in the therapeutic performance, target specificity, control released and overcoming of drug resistance.

In this study, the approach to developing new carrier systems moved to a different level using two different chitosan phyto-nanocarriers from hellebore. The first nanocarrier involved the encapsulation of hellebore in chitosan nanoparticles. The second one emerged in a new phyto-carrier system based on AgNPs encapsulated in chitosan nanoparticles to ensure the complementary and synergistic action of AgNPs and the secondary metabolites from hellebore. To the best of our knowledge, this is the first study reporting on the concurrent use of hellebore, chitosan and AgNPs. The physical, chemical and antioxidant properties of the new nanocarriers were evaluated.

## 2. Results and Discussion

Plants encompass a multitude of secondary metabolites with highly complex chemical structures. There is a close relationship between environmental factors and the configuration and proportion of bioactive compounds in a specific plant [40,41]. Furthermore, the amount of active phytoconstituents depends on the selected experimental conditions (extraction procedure, temperature, time, etc.) [4,11,42].

The collective action of all secondary metabolites dictates the biological activity of a particular plant [43,44]. Consequently, determining a direct connection between the chemical composition of a plant and its therapeutic activity is arduous. Additionally, the physical–chemical properties (size, chemical or thermic stability, etc.) of some phytochemicals isolated from plant material impose restrictions on the biological environment (e.g., biodisponibility) and, thus, significantly diminish their bioefficiency [45].

Studies on Romanian *Helleborus purpurascens* are few and target only the content of a few classes of phytochemicals (solely isolated from the plant root) and their pharmacological activities [5,6,7,8,9,10,11]. In addition, the integrated chemical screening of several plant parts (stem, leaves, and flowers) of this medicinal plant from Romanian natural vegetation has not been carried out yet. The secondary metabolite chemical screening of hellebore samples was performed using gas-chromatography coupled with mass spectroscopy (GC-MS) and electrospray ionization-quadrupole time-of-flight mass spectrometry (ESI-QTOF-MS) analysis.

GC-MS is one of the most accurate and fast techniques used for analyzing various phytochemicals with relatively low molecular mass separation and to confirm identification [46]. To this end, the separation of the secondary metabolites from the hellebore samples was investigated (Figure S1 and Table 1)

The results of the tentative compound identification from the hellebore sample using the GC-MS method are shown in Table 1.

The GC-MS analysis (Figure S1) indicated that eight bioactive phytoconstituents were separated (96.75% of the total peak area), taking into consideration that additional procedural methods conducted before the GC-MS analysis (for instance, derivatization) are requested thermally unstable phytochemicals [46].

Conversely, the electrospray ionization-quadrupole time-of-flight mass spectrometry (EIS-QTOF-MS) analyzer allows for the fast, affordable and highly accurate identification of secondary metabolites from complex matrices. To this end, the hellebore’s metabolite profile screening was performed using the mass spectrometry method. The mass spectra depicted a sophisticated mix of low-molecular-weight biomolecules, of which some were detected and compared for identification with those of the NIST/EPA/NIH Mass Spectral Library 3.0 database and in the literature review [2,3,4,6,7,8,9,10,11].

The mass spectrum and the hellebore’s secondary metabolites identified in the ESI–QTOF–MS analysis are presented in Figure S2 and Table 2, respectively.

### 2.1. Screening and Classification of the Differential Metabolites

A total of 105 secondary metabolites identified through mass spectroscopy were assigned to different chemical classes: amino acids and peptides (60.95%), terpenoids (6.67%), fatty acids (6.67%), flavonoids (5.71%), phenolic acids (4.76%), bufadienolides (3.80%), sterols (1.90%), glycosides (0.95%), alkaloids (0.95%), phytoecdysteroids (0.95%), nucleosides (0.95%), butenolides (0.95%), carbohydrates (0.95%) and miscellaneous. The assignment of the identified phytoconstituents into different chemical categories is presented in Table 2.

**Figure 1:**
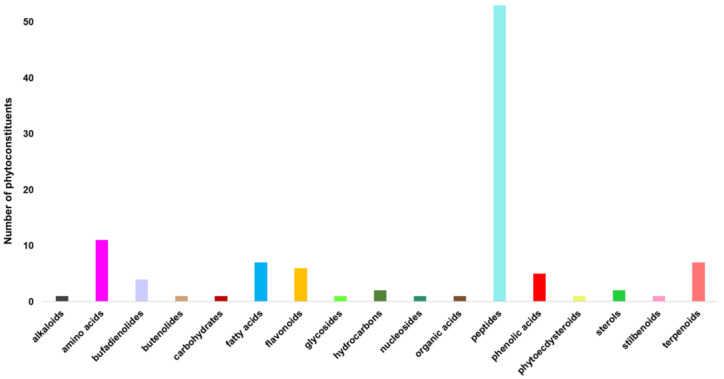
Phytoconstituent classification bar chart for *Helleborus purpurascens*.

According to Figure 1, amino acids and peptides are the largest categories of phytochemicals, constituting about 60.95%. An equal proportion of essential amino acids (phenylalanine, isoleucine, tryptophan, threonine and histidine) and nonessential amino acids (alanine, ornithine, glutamic acid, cysteine and glycine) were found in the hellebore sample. A large percentage (over 70%) of this category of secondary metabolites (isoleucine, tryptophan, phenylalanine, arginine, histidine, glycine, alanine and glutamic acid) exhibit cytotoxic, antiproliferative and immunomodulating activity [65,66,67,68,69,70,71].

Over 50 small peptides (di-, tri- and tetrapeptides) were identified in the hellebore sample. Over two-thirds of these are sulfur-containing peptides. Various studies have reported on the antitumoral, antimicrobial, neuroregulating and antiviral (anti-HIV) properties of phytopeptides and thionins [68,72,73,74,75]. This difference in the compositions of the small peptides in the hellebore samples compared to our previously reported results [11] confirms that the profile of the secondary metabolites is determined by various endogenous or ecological factors, plant components, extraction methods and solvent polarities [40,41,42,76,77,78].

*Bufadienolides* are a category of metabolites with remarkable antitumoral, antiviral and anti-inflammatory properties [79,80,81]

*Butanolides* (anemonin) act as anti-inflammatory and antimicrobial agents [82,83].

*Phytoecdysteroids* are a category of secondary metabolites with notable antitumoral, antimicrobial, antiviral, anti-inflammatory, antioxidant, hepatoprotective and antidiabetic activities [84].

*Terpenoids and sesquiterpenoids* are another category of phytochemicals that were found in the hellebore samples. Numerous studies have demonstrated their notable therapeutic properties: antimicrobial, antioxidant, anticonvulsant, analgesic, neuroprotective, anti-inflammatory, anti-allergic and antitumor [66,85,86,87,88,89]. Furthermore, protoanemonin exerts antimicrobial and anti-HIV effects [2,90,91].

*Fatty acids* represent approximately 7% of the phytochemicals identified in the hellebore samples. Numerous research has reported on their antioxidant, anti-inflammatory, neuroprotective and cardiovascular protective properties [66,89,92].

*Flavonoids* are secondary metabolites with outstanding pharmacological effects: antioxidant, anti-inflammatory, antimicrobial, antitumoral and neuroprotective [93,94,95,96].

*Phenolic acids* represent 4.7% of the biomolecules identified in the hellebore samples. Extensive studies have demonstrated their significant therapeutic properties (antioxidant, antimicrobial, antimicrobial, antitumoral, neuroprotective and antidiabetic) [96,97,98].

*Phytosterols* are steroids have antioxidant, antitumoral, anti-inflammatory, immunomodulatory, neuroprotective, antidiabetic, cardiovascular protective and osteoporosis protection properties [99].

*Carbohydrates* act as antioxidant, anti-inflammatory, antibacterial, antiviral, antitumoral, immunomodulatory, antidiabetic and cardioprotective agents [65,100,101].

*Glycosides* are biomolecules with notable antitumoral (leukemia and gastric tumors) properties [102].

Magnoflorine, the *alkaloid* identified in the hellebore samples, has shown antitumoral, neuropsychopharmacological, immunomodulatory, anti-inflammatory, antioxidant and antifungal activities [103].

The *nucleoside* uridine act as a neuroregenerative and neuroprotective agent [104].

Among the *miscellaneous* compounds identified in the hellebore samples, resveratrol has antioxidant, antitumoral anti-inflammatory, immunomodulatory, antiobesity, antidiabetic, neuroprotective and cardiovascular protective effects [105].

### 2.2. Phyto-Nanocarriers

Secondary metabolites from plants have excellent therapeutic properties. However, most of these biomolecules exhibit reduced bioavailability in vivo and an inability to cross biological membranes because of their physicochemical properties (size, chemical stability, polarity, etc.) [12,68,106,107,108]. 

The foremost hurdles of the current antitumoral therapy are target-specificity, retention and permeability, and need to minimize the side effects and overcome chemoresistance [12,68,108]. Therefore, the development of cutting-edge phyto-nanocarrier systems through hellebore’s encapsulation in chitosan nanocapsules (HCs) and a hellebore-AgNP system’s entrapment in chitosan nanocapsules (HAgCs) combines the biological activity of phytoconstituents and a natural biopolymer in the case of the first phyto-nanocarrier. In addition, the second photo-nanocarrier (HAgC) is added to the silver nanoparticles’ biological activity to achieve a new therapeutic strategy that could overcome the resistance of bacteria to antibiotics or the drug resistance of cancer cells. Furthermore, this new approach increases the target specificity, precise control release, stability and permeability.

### 2.3. FT-IR Spectroscopy

FT-IR is an essential analytical tool that is widely used for the rapid, efficient and sensitive analysis of multicomponent systems [109].

**Figure 2:**
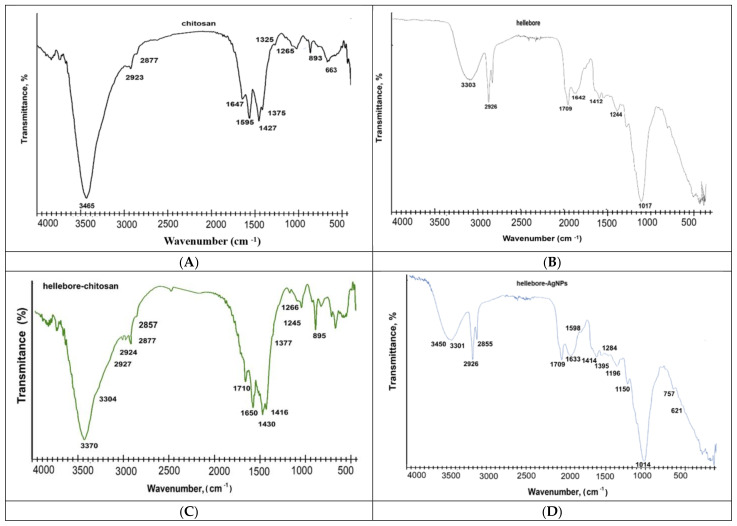
FT-IR spectra: (**A**) chitosan; (**B**) hellebore; (**C**) HC phyto-nanocarrier; (**D**) hellebore-AgNPs system.

**Figure 3:**
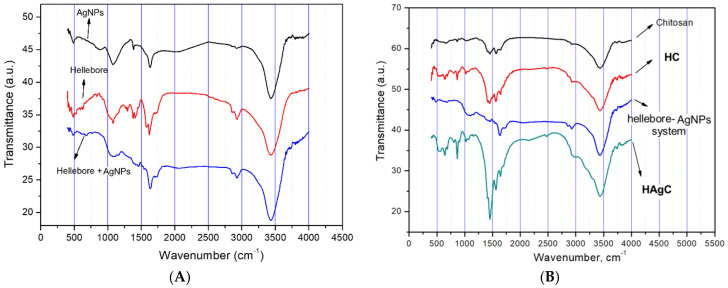
FT-IR spectra: (**A**) citrate-coated AgNPs, hellebore and hellebore-AgNPs; (**B**) chitosan, HC, hellebore-AgNPs system and HAgC.

The first phyto-nanocarrier’s (HC) preparation and the following preparation of the second phyto-nanocarrier occurred in two stages: (a) the metallic nanoparticles were incorporated into the pores of the hellebore; (b) the encapsulation of the hellebore-AgNPs into the chitosan nanoparticles was investigated using FT-IR spectroscopy to identify the functional groups specific to each component. The individual FT-IR spectrum of the hellebore, chitosan, citrate-coated AgNPs, hellebore-AgNPs system, and HC and HAgC phyto-nanocarrier(s) are shown in Figure 2 and Figure 3**.** The FT-IR absorption bands identified in the hellebore samples are presented in Table 3.

The FT-IR peak of the AgNPs coated with trisodium citrate (surfactant) (Figure 3A) presented vibrational bands characteristic of a surfactant at 3435 cm^−1^ (H-OH stretching vibration) and 2927 cm^−1^, as well as at 2852 cm^−1^ (CH- asymmetric and symmetric stretching vibrations), 1631 cm^−1^ (COO- stretching vibration) and 1387 cm^−1^ (C-H bending) [130].

The FT-IR peak of the chitosan (Figure 3B) showed characteristic vibrational bands at 3363 cm^−1^ (associated to N-H and O-H stretching); 2923 and 2877 cm^−1^ (attributed to C-H stretching); 1647 cm^−1^ (corresponding to the C=O stretching of amide); 1427 and 1375 cm^−1^ (associated to C-H bending); 1325 cm^−1^ (associated to a tertiary amide); 1265 cm^−1^ (corresponding to C-OH vibration); 890 cm^−1^ (attributed to C-H bending); and 663 cm^−1^ (associated to N-H vibration) [131,132,133,134].

Regarding the spectra of the HC phyto-nanocarrier (Figure 3B), all of the characteristic peaks of chitosan (2924, 2877, 1650, 1430, 1377, 1266 and 890 cm^−1^) and the plant phytoconstituents were observed. Nevertheless, the intensity of the band changes in the C-O and C-H regions (890 and 1380 cm^−1^) indicated the encapsulation and, probably, the hydrogen bond formation between the hellebore and chitosan [135].

The FT-IR spectra of the hellebore-AgNPs system (Figure 3A) included the characteristic peaks of hellebore at 3383 cm^−1^, attributed to the -OH group; at 2928 cm^−1^, assigned to C-H aromatic; at 1742, assigned to δ-lactone C=O and the lactone ring of bufadienolides; at 1709, assigned to carboxyl acid C=O stretching; at 1653, assigned to the β-unsaturated keto group and ecdysteroids; at 1642, corresponding to amino acids group N-H; at 1244 and 1017, attributed to the C-N of amine; at 881.34 and 815 cm^−1^, assigned to C-O and CH vibrations of aromatic rings, as well as AgNPs coated with surfactant (trisodium citrate) [130]. In addition, the absorption bands at 1635, 1388, 1115 and 676 cm^−1^ that appeared in the synthesized AgNPs solution (Figure 3B) shifted to higher wavenumbers (1639.7, 1428.3, 1151 and 683 cm^−1^), suggesting the binding of AgNPs to the O–H, C=O, N-H and C–O functional groups of the hellebore phytoconstituents (Figure 3 and Table 3) [130,136]. Moreover, several detectable changes occurred in the hellebore spectra, in particular, in the case of the hydroxyl vibrations (O-H stretching (the absorption bands at 1575 (N-H), 1415 and 1382 (O-H), and 1294, 1267 and 1081 (C-O)) which shifted to lower wavenumbers, indicating that this functional group is involved in the binding of AgNPs (Figure 3 and Table 3). Based on the analysis of the FTIR spectra of the HAgC phyto-nanocarrier (Figure 3B), it appeared to exhibit the characteristic vibrational bands of the chitosan sample and hellebore-AgNPs system with small shifts. A pronounced sharpening of the O–H and N-H stretching regions in the FT-IR supports the preparation of the HAgC phyto-nanocarrier. Additionally, these changes in the band intensity indicate that this functional group is involved in the binding of the hellebore-AgNPs system, probably via hydrogen bonding [135]. The FT-IR data for both of the phyto-nanocarriers’ development (HC and HAgC) is important for understanding their behavior and can aid in their further analysis.

### 2.4. X-ray Diffraction Spectroscopy

The XRD analysis provided valuable insight into the crystalline nature of the samples, the degree of order and the arrangement of the atoms in the crystal lattice of the initial and final products.

**Figure 4:**
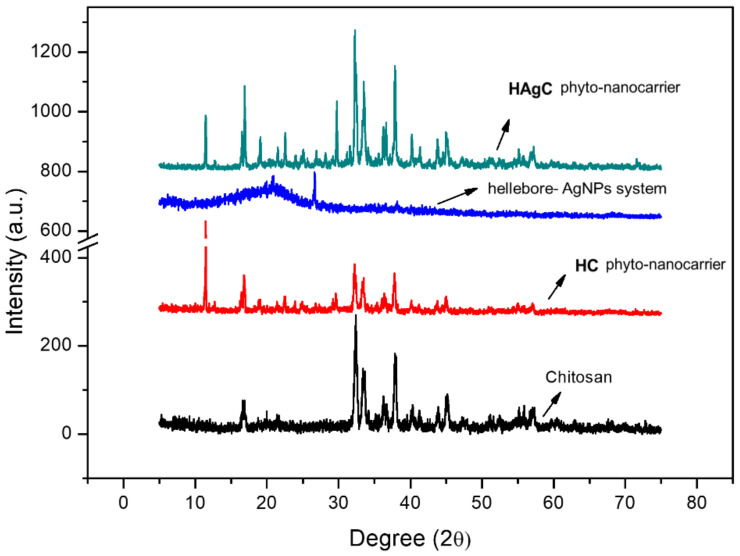
Powder XRD patterns of the CN, hellebore-AgNPs system, and HC and HAgC phyto-nanocarriers.

Figure 4 shows the XRD patterns of the chitosan, hellebore-AgNPs system, HC and HAgC phyto-nanocarriers. The chitosan exhibited peaks at 2θ: 11.55°, 16.37°, 18.33°, 22.4° and 34.4°, indicating the crystalline form II structure of chitosan [137,138,139].

The XRD pattern for the hellebore-AgNPs system presented a mix of crystalline and amorphous patterns with peaks at 2θ: 15.82° (weak), 22.23°, 27.87° and 38.15°.

The peaks at 15.82° (weak) and 22.23° (strong) are associated with hellebore constituents. In addition, the two distinct diffraction peaks of the 2θ: values of 27.87°and 38.15°, as well as 64.4° and 78.5°, indicate the crystalline structure of the AgNPs [39,139], while the specific XRD spectrum of the HC phyto-nanocarrier displays characteristic peaks of chitosan (at 2θ: 11.55°, 16.37°, 18.33°, 22.4° and 34.4°) and hellebore (at 2θ: 15.82° and 22.23°), which are considerably attenuated. Accompanying the XRD pattern for the HAgC phyto-nanocarrier, a few differences were found, despite the similarities to that of hellebore and chitosan. For example, the characteristic bands of the plant at 2θ = 15.8° and 22.3° were slightly weaker, and the AgNP peaks can easily be observed at 2θ = 38.2°, 44.1°, 64.4° and 78.5°.

The XRD results demonstrate that the new phyto-nanocarriers were successfully prepared through encapsulation into chitosan nanoparticles [140,141]. The mean crystallite size obtained via XRD analysis for CN (18.87 nm), HC (21.23 nm) and HAgC (23.66 nm) were confirmed again using SEM.

### 2.5. Scanning Electron Microscopy (SEM)

**Figure 5:**
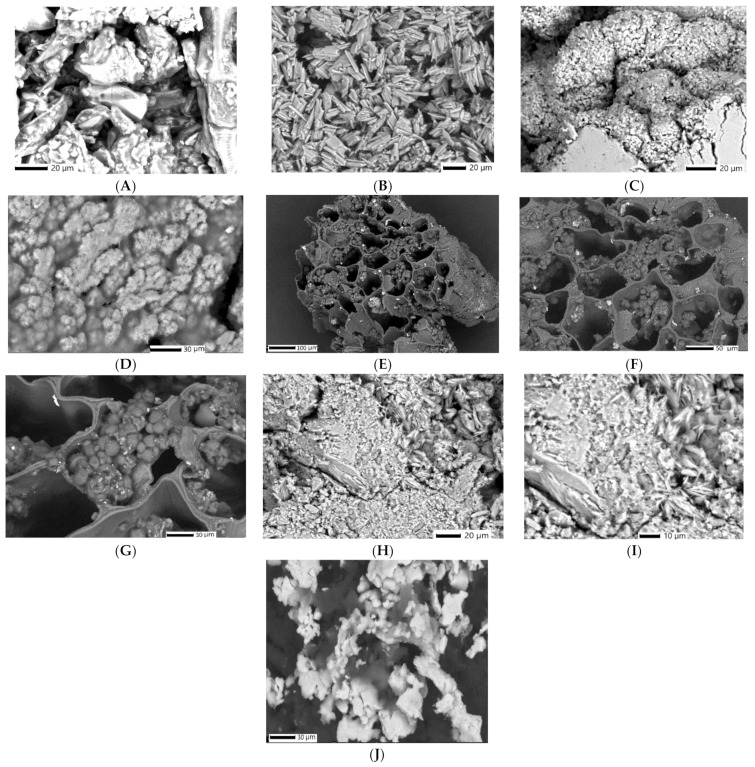
SEM images: (**A**) hellebore; (**B**) chitosan; (**C**,**D**) HC phyto-nanocarrier; (**E**–**G**) hellebore-AgNPs system; (**H**–**J**) HAgC phyto-nanocarrier.

The surface morphology, shape and particle size of both phyto-nanocarriers (i.e., HC and HAgC), as well as their components (i.e., hellebore and chitosan) were studied with SEM (Figure 5).

The hellebore micrograph (Figure 5A) exhibits a porous, irregular structure. The morphology of the chitosan particles (Figure 5B) indicates the presence of clusters of rod-shaped crystals (~20 nm). The HC phyto-nanocarrier micrographs (Figure 5C,D) each show a spherical cluster of nanoparticles. A notable difference is apparent, particularly in terms of the particle size (~21.2 nm). Another visible aspect is the presence of numerous uniform spherical-shaped nanoparticles. The morphology of the hellebore-AgNPs system (Figure 5E–G) indicates the changes in the hellebore that appeared after the preparation of the hellebore-AgNPs system. A noticeable element is the presence of numerous uniform, nanosized silver particles (~28 nm) loaded in the porous structure, which has an irregular shape and is the same size as the hellebore particles. After encapsulation, SEM images of the HAgC phyto-nanocarrier (Figure 5I–J) show the appearance of clusters of nanospherical particles (~23.5 nm). Additionally, the SEM (Figure 5C,D,H–J) analysis indicated a modification of the shape, a decrease in the size of the particles and a tendency toward agglomeration for both of the encapsulated phyto-materials (hellebore and phyto-carrier system). These changes in the morphology suggest that under the employed experimental conditions, the preparation of the HC and HAgC phyto-nanocarriers was successfully achieved [142].

**Figure 6:**
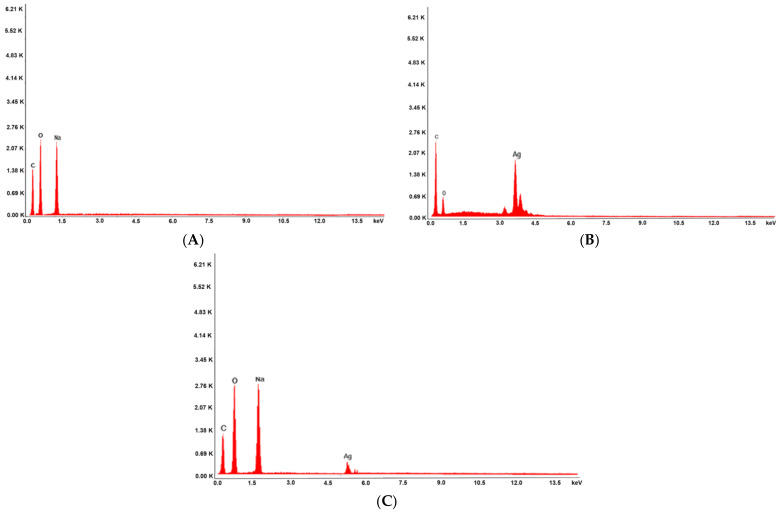
EDX composition: (**A**) HC phyto-nanocarrier; hellebore-AgNPs system (**B**) before and (**C**) after encapsulation.

Accompanying the SEM spectra were EDX analyses on the elemental composition of the HC, hellebore-AgNPs system and HAgC samples investigated (Figure 6).

The EDX spectra of the HC phyto-nanocarrier indicated the presence of both hellebore and chitosan components (Figure 6A). Analogous work with the hellebore-AgNPs system indicated the introduction of AgNPs in the hellebore’s pores (Figure 6B). An elemental analysis of the HAgC phyto-nanocarrier was conducted using EDX spectroscopy (Figure 6C). It appears that there were differences compared to the EDX spectra before encapsulation, thus, confirming the HAgC phyto-nanocarrier’s preparation.

### 2.6. Dynamic Light Scattering

Dynamic light scattering (DLS) is a fast, simple and accurate method that is widely used to determine the size and distribution profile of nanoparticles suspensions. 

**Figure 7:**
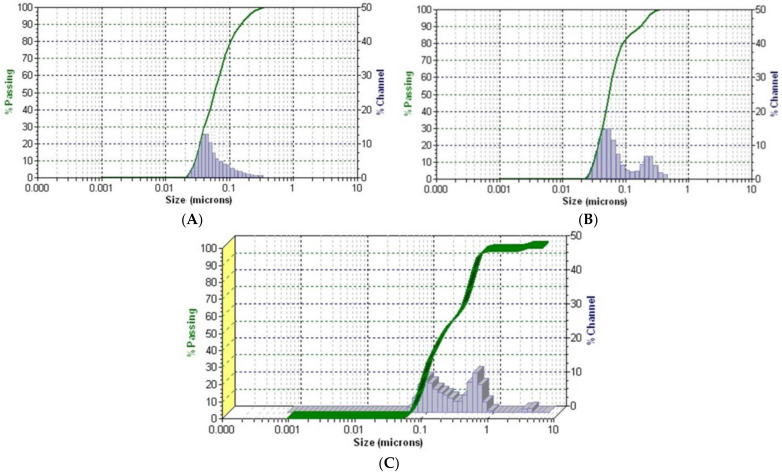
DLS patterns: (**A**) chitosan particles; (**B**) HC and (**C**) HAgC phyto-nanocarriers.

In this study, the DLS method was employed to determine the average mean of each phyto-nanocarrier prepared and their raw components (Figure 7).

The results indicate a mean hydrodynamic diameter for the chitosan sample of 100.2 nm (Figure 7A). The DLS pattern the HC phyto-nanocarrier (Figure 7B) exhibited two peaks that can be attributed to chitosan and hellebore nanoparticles. Moreover, the HAgC phyto-nanocarrier (Figure 7C) results showed an additional peak that can be attributed to the AgNPs. Nonetheless, a notable difference appeared, specifically in the intensity peaks of the HC and HAgC phyto-nanocarriers, suggesting the formation of an agglomerate. Furthermore, the increase in the sample’s diameter size to between the values determined via the XRD and SEM analyses could be attributed to the manner in which the particles interacted with each other in the suspension (i.e., ultrapure water). In addition, various studies have reported that a shift toward larger average sizes is associated with encapsulation [131,143]. Another visible aspect was the distribution of particles in a narrow range, demonstrating the good stability of the newly prepared phyto-nanocarriers.

### 2.7. Thermal Behavior Study

**Figure 8:**
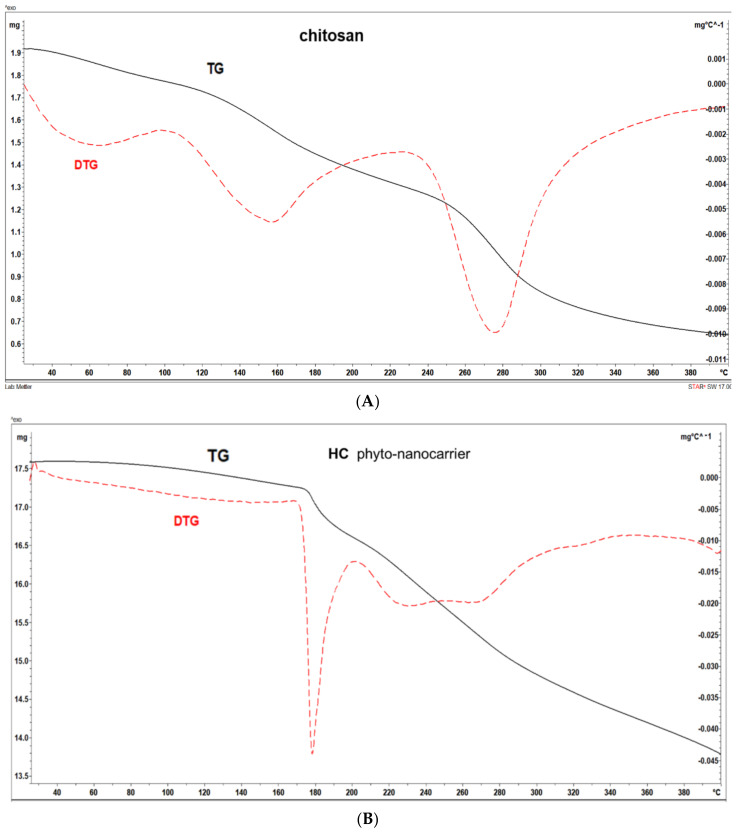

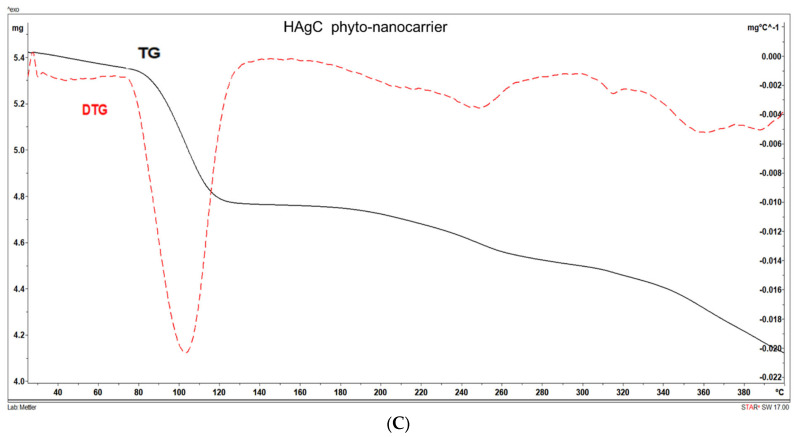
TG/DTG thermograms: (**A**) chitosan sample; (**B**) HC; (**C**) HAgC.

**Figure 9:**
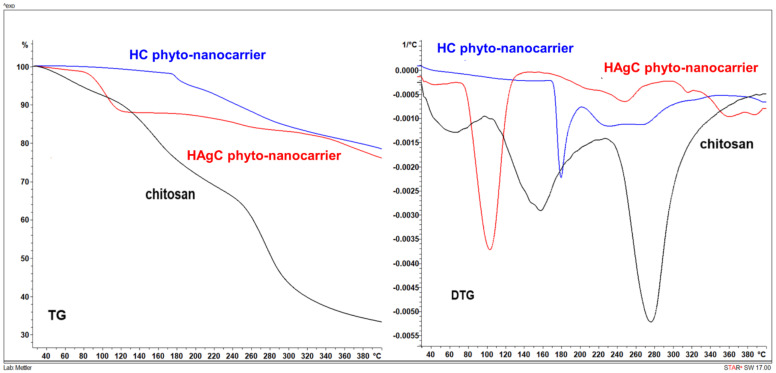
Comparison of the thermal stability of both phyto-nanocarriers (HC and HAgC).

The thermogravimetric analysis (TG)/differential thermogravimetric *analysis* (DTG) method was used to investigate the thermal properties of the chitosan matrix and HC and HAgC phyto-nanocarriers. The results are presented in Figure 8 and Figure 9.

According to Figure 8A and Figure 9, the chitosan followed three thermal decomposition steps in the temperature range of 25–400 °C. The first weight loss at a temperature up to 100 °C corresponds to the loss of adsorbed water. The second loss at a temperature range of 100–230 °C was due to the onset of chitosan decay (i.e., pyrolysis of the polysaccharide structure), which begins with a cleavage of glycosidic bonds. The final stage of weight loss occurred at temperatures above 230 °C and can be attributed to the advanced decomposition of polysaccharides with the formation of acetic and butyric acids and a series of lower fatty acids [144]. Chitosan’s degradation was not completed in the studied temperature range.

The results for the HC phyto-nanocarrier (HC) (Figure 8B and Figure 9) exhibited a minimum of weight loss at temperatures up to 100 °C, which was related to moisture evaporation. Then, the decomposition of the HC compound occurred in two stages of decomposition represented by separate thermoanalytical curves. The first occurred in the range of 170–200 °C, reaching a maximum of 178 °C, and showed a weight loss of 3.58%. The second weight loss (8.19%) step involved a complex process and took place in the temperature range between 200 and 278 °C. The higher weight loss (19.68%) in the temperature range of 170–400 °C can be attributed to chitosan’s degradation and, to a smaller extent, its deacetylation [135].

Regarding the HAgC nanocarrier, the TG/DTG curve (Figure 8C and Figure 9) followed the thermal trends of its components (i.e., chitosan and hellebore), with several exothermic decomposition steps in the temperature range of 70–380 °C.

**Figure 10:**
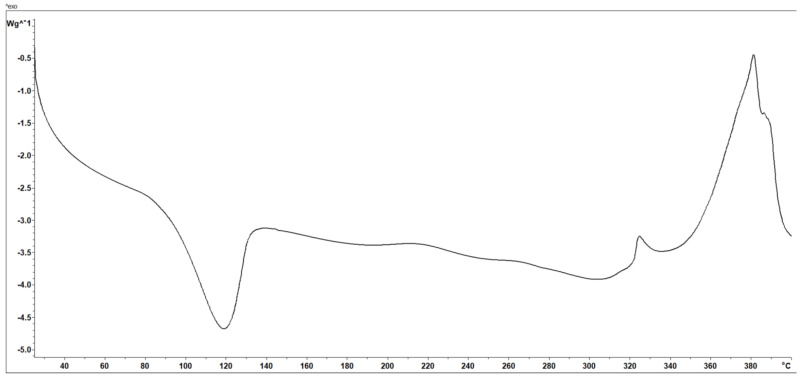
DSC curve of the HAgC phyto-nanocarrier.

The first weight loss, at temperatures up to 70 °C, corresponded to the evaporation of adsorbed water. The higher weight loss (10.76%) occurred in the temperature range of 73–126 °C, followed by a continuous loss of mass with stages that are problematic to separate. Nonetheless, four maxima on the DTG curve were present. This behavior could be attributed to supplementary groups and a higher hydroxyl group number from the encapsulated hellebore-AgNPs in the chitosan matrix [135,145]. The DSC analysis of the HAgC phyto-nanocarrier is presented in Figure 10.

The data reveal an endothermic process with a maximum of 119 °C and two exothermic processes at 328 °C and 380 °C. The thermic profile of the HAgC phyto-nanocarrier showed a sharp endothermic peak attributed to the various water-holding capacities and interaction strengths between the water and polymer. In addition, the exothermic peaks reflected the chitosan’s degradation (glycosidic ring dehydration, depolymerization and chemical decomposition of the monosaccharide units) [146,147,148,149]. The changes in the DTG thermograms observed for the HC and HAgC phyto-nanocarriers (Figure 9) (compared to the chitosan sample (shift of the endothermic effects) correspond to the interaction between chitosan and the hellebore and hellebore-AgNPs system, respectively. These data point to the (i) successful preparation of the HC and HAgC phyto-nanocarriers into chitosan nanoparticles and (ii) the overall improved thermal stability of these materials. These results corroborate those reported in the literature [146,149,150,151].

### 2.8. Screening of the Antioxidant Activity

The highly complex composition of bioactive compounds from a specific herb imposes the selection of several assays for the evaluation of the antioxidant capacity, since their separation and quantification are arduous because of their chemical diversity [37]. Therefore, methods for the direct estimation of the total antioxidant activity potential from plant matrices are preferred [37,38,152,153]. There are several chemical and biochemical antioxidant capacity assays. The most commonly used in vitro tests include the hydrogen atom transfer (HAT) and electron transfer (ET) methods. HAT methods include ORAC, TRAP and TOSCA, among others, while the primary ET methods include the Folin–Ciocalteu reagent assay, DPPH, TEAC, FRAP, and others [37,38,152,153]. The selection of the most appropriate method depends on a diverse set of factors, like simplicity, sensitivity, cost and reproducibility. To gain a comprehensive understanding of a plant’s antioxidant potential [37,38,152,153,154,155], and, therefore, for a more accurate assessment of the antioxidant potential of hellebore microencapsulated in chitosan and the new phyto-carrier system in vitro, noncompetitive methods—DPPH, o-phenanthroline (OPM), Folin–Ciocalteu and phosphomolybdate (total antioxidant capacity)—were chosen.

### 2.9. DPPH (1,1-Diphenyl-2-Picrylhydrazyl) Free Radical Scavenging Assay

**Figure 11:**
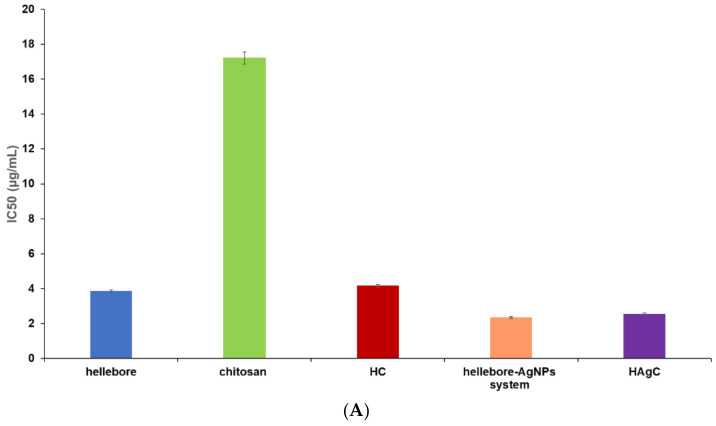

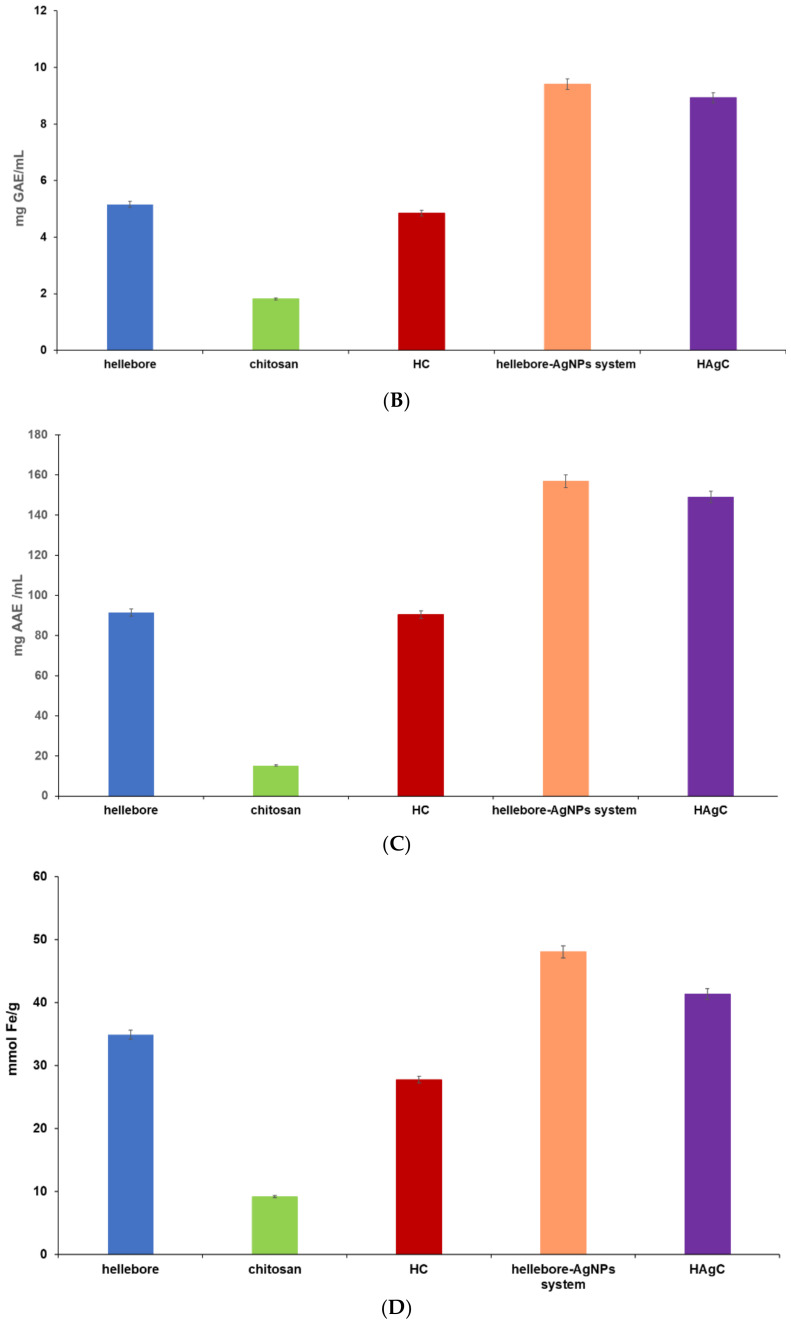
Graphic representation: (**A**) DPPH results expressed as IC_50_ (mg/mL); (**B**) total polyphenolic contents assay results; (**C**) phosphomolybdate (total antioxidant capacity) results; (**D**) iron(III)-phenanthroline assay results.

The DPPH assay is one of the simplest, most accurate and cheapest methods based on a single electron transfer (ET) for evaluating the antioxidant capacity of complex plant matrices. It is based on the stable, free radical DPPH, which acts as a hydrogen donor [35,37,39,153,156]. Hence, the antioxidant activity of the new carrier(s) and its components were evaluated using this technique. The data obtained are presented in Table 4 and Figure 11A.

The IC50 values obtained indicate a significant increase in the antioxidant activity of the hellebore-AgNPs system correlated with the plant sample. Thus, the IC50 value was lower by over 60% than that of the hellebore samples, attributed to the synergistic action of the AgNPs and the phenolic compounds in the hellebore. Concomitantly, there is a slight decrease in the antioxidant activity of both prepared phyto-nanocarriers compared to the hellebore samples and the hellebore-AgNPs system. The increase in the IC50 values for the HC by 7% and HAgC by almost 8% compared to the nonencapsulated components might be due to the protective effect of the chitosan nanocapsules, which partially limits direct contact with the DPPH reagent, which is in good accordance with the results reported in the literature [143,157].

The chitosan sample presented relatively low scavenging activity against DPPH, and the IC50 value obtained was close to that reported in the specialized literature [158,159].

### 2.10. Total Polyphenolic Contents (TPCs) Assay—Folin–Ciocalteu

The Folin–Ciocalteu assay is a classic electron transfer (ET) technique used to establish the total phenol/polyphenol content from complex vegetable matrices or from food [39,153,160,161].

The total polyphenolic contents (TPCs) of the new phyto-nanocarrier(s) and its raw materials were determined, and the obtained results are presented in Table 4 and Figure 11B.

According to the data obtained, with the first phyto-nanocarrier, HC, more than 94% of the polyphenols in the hellebore sample reacted with the Folin–Ciocalteu reagent due to the density of the chitosan layer [151]. The antioxidant activity of the HAgC represented approximately 95% that of the hellebore-AgNPs system’s because of the synergistic action of the AgNPs, with a catalytic role played by the hellebore’s polyphenolic compounds. As reported in the literature [157], this slight decrease in the antioxidant activity detected for both phyto-nanocarriers indicates the good encapsulation efficiency of the bioactive compounds. The total phenolic content value determined for the chitosan sample is in good agreement with the literature [162]. 

### 2.11. Phosphomolybdate Assay (Total Antioxidant Capacity)

Phosphomolybdate (total antioxidant capacity) is a simple, sensitive and common method to determine the total antioxidant potential of plant extracts or other complex mixtures of biomolecules. The assay is based on the molybdenum (VI) to molybdenum (V) reduction through an ET or HAT mechanism [163].

The phosphomolybdate assay (total antioxidant capacity) was used to determine the total antioxidant potential of the prepared phyto-carrier system and hellebore encapsulated in chitosan compared to those of the hellebore, hellebore-AgNPs, chitosan and ascorbic acid. The obtained experimental results are displayed in Table 4 and Figure 11C.

Table 4 indicates that both phyto-nanocarriers displayed a slight decrease in antioxidant activity (less than 2–5%) than the nonencapsulated components suggesting that, under the employed experimental conditions, the chitosan matrix protected the core bioactive compound and also acted as an antioxidant activity booster agent [154]. The superior antioxidant activity of the hellebore-AgNPs complex compared to the plant sample can be attributed to the synergistic and complementary action of the secondary metabolites from the hellebore and AgNPs [164,165].

### 2.12. Iron(III)-Phenanthroline Antioxidant Assay

The o-phenanthroline method (OPM) is a simple, fast, sensitive and accurate method for hydroxyl radical scavenging activity evaluation in complex samples. The assay is based on the inhibition of the formation of Fe (II)-1,10-phenanthroline using hydroxyl radical as a quantitative measure of the scavenging activity [163,166,167,168,169,170].

The iron(III)-phenanthroline antioxidant assay was used to measure the antioxidant activity of the new phyto-nanocarriers and their components. The results are presented in Table 4 and Figure 11D.

The hellebore-AgNPs system exhibited a higher antioxidant activity than the hellebore sample (more than 70%) due to the complementary action of the bioactive compounds and the antioxidant mechanism of the AgNPs (quenched free radicals by donating or accepting electrons) in the presence of the hellebore’s various phytoconstituents. Additionally, studies have reported that AgNPs’ structural features (i.e., shape and size) affect antioxidant activity [130,171]. In the case of phyto-nanocarriers, there was a decrease in the antioxidant activity (about 20% for HC and almost 14% for HAgC), which indicates that the encapsulation was successful, and the chitosan matrix prevented the complete release of all antioxidant agents from the core phyto-materials [157].

Altogether, the evaluation of both phyto-nanocarriers’ antioxidant potential through the four assays shows a slight decrease compared to the nonencapsulated components. However, the antioxidant activity of the two nanocarriers is still very high, considering that their antioxidant performance depends on the physicochemical characteristics of chitosan and the experimental conditions (reagent ratio, pH, temperature, solvent polarity, etc.) [172]. The collective results indicate the success of the encapsulation and, thus, an enhancement of the in vivo biocompatibility and stability, as well as prolonged controlled-release [143,157,173].

### 2.13. Encapsulation Efficiency, Loading Capacity and Encapsulation Yield (EY%)

The encapsulation efficiency, loading capacity and encapsulation yield are the determining parameters for establishing the quality of nanocapsules and their application potential and economic feasibility [145,174].

The data obtained are shown in the Table 5.

According to Table 5, the encapsulation yield decreased in the following order: HAgC > HC > CN.

These results indicate that the hellebore and hellebore-AgNPs were efficiently entrapped in the chitosan nanoparticles, which can be attributed to electrostatic interactions between the positively charged (amino) groups in the chitosan nanoparticles and the negatively charged groups of the different phytoconstituents from the hellebore sample [139,175,176].

Moreover, the higher values for the encapsulation parameters in the case of the HAgC compared to the HC indicate the occurrence of various interactions (electrostatic, hydrogen bonds, Van der Waals) between the amino groups of the chitosan matrix and the AgNPs [177].

## 3. Materials and Methods

All reagents used in this study were of analytical grade, purchased from commercial sources and used without further purification. Ethanol, methanol, chloroform, dichloromethane, FeCl_3_·6H_2_O, FeSO_4_·7H_2_O and anhydrous Na_2_CO_3_ were from Sigma Aldrich (München, Germany). 1,10-Phenantroline; Folin–Ciocalteu phenol reagent (2 N); 2,2-diphenyl-1-picrylhydrazyl reagent; gallic acid; ascorbic acid; AgNO_3_; sodium citrate; sodium carbonate; potassium persulfate; sodium phosphate; ammonium molybdate; and potassium chloride of 99% were also purchased from Sigma-Aldrich (München, Germany). Propyl gallate (purum) was purchased from Fluka (Buchs, Switzerland). Chitosan (MW 100,000–300,000) was purchased from Acros Organics (Verona, Italy). Ultrapure water (DDW) was used throughout the experiments.

The *Helleborus purpurascens Waldst. & Kit*. samples (whole plant: stem (28 cm in height), leaves, roots and rhizome (8 cm)) were harvested in March 2023 from an area in Prahova County, Romania (geographic coordinates: 45°20’46.4689750856423” N, 25°33’22.9191970825194” E), and taxonomically authenticated at the University of Medicine and Pharmacy Craiova, Romania.

### 3.1. Phyto-Carrier-System Components’ Preparation

The AgNPs’ synthesis was carried out according to a procedure described in our earlier publication [130].

#### Plant Samples’ Preparation for Chemical Screening

The plant samples were chopped and frozen in liquid nitrogen (−185 °C). Subsequently, they were milled using a planetary mill (Fritsch Pulverisette mill, Idar-Oberstein, Germany), sieved to obtain particle diameters ranging from 0.30 to 0.35 mm and then stored at −30 °C to avoid the phytoconstituents’ enzymatic degradation. The ultrasonic-assisted extraction’s experimental procedure consisted of the following: round-bottom flasks (50 mL) containing 1.8 g dried plant sample and 18 mL of solvent (methanol/chloroform = 1:1) were kept at 40 °C and 50 Hz for 35 min (Elmasonic, Singen, Germany). The solvent was evaporated at 40 °C using a rotary evaporator (Rotavapor; BÜCHI, Switzerland), followed by the dissolution of the residue obtained with MeOH (10 mL), filtration through a 0.2 µm syringe filter and storage at −30 °C until further analysis. All experiments were prepared in triplicate.

### 3.2. GC-MS Analysis

Gas chromatography was carried out on a GCMS-QP2020NX Shimadzu apparatus with a ZB-5MS capillary column (30 m × 0.25 mm id × 0.25 µm) (Agilent Technologies, Santa Clara, CA, USA), helium, and flow of 1 mL/min.

#### GC–MS Separation Conditions

The oven temperature was increased from 50 °C (held for 2 min) to 300 °C at a rate of 6 °C/min (held for 5 min). The temperature of the injector was 280 °C, and the temperature at the interface was 220 °C. The mass of the compounds was registered at a 70 eV ionization energy. The mass spectrometer was source-heated at 230 °C, and the MS Quad was heated at 150 °C. Compounds were identified based on their mass spectra, which were compared to the NIST 3.0 database mass spectra library database (USA National Institute of Science and Technology software) (NIST, Gaithersburg, MD, USA). Retention indices (RIs) were also calculated for each compound based on a C_8_–C_20_ alkane standard mixture calibration curve, which was compared to Adams indices in the literature [178].

A percentage area of each compound was calculated by summing all of the experimental integrated peak areas (Table 1).

### 3.3. Mass Spectrometry

The MS experiments were performed using an EIS-QTOF-MS (Bruker Daltonics, Bremen, Germany). The mass spectra were acquired in the positive ion mode in a mass range of 40–3000 *m*/*z*. The scan speed was 2.0 scans/s, the collision energy was 10–85 eV, and the temperature of the source block was 82 °C. The standard library NIST/NBS-3 (National Institute of Standards and Technology/National Bureau of Standards) (NIST, Gaithersburg, MD, USA) was used for their assignment to specific bioactive compounds. The results are presented in Table 2.

#### 3.3.1. Phyto-Carrier System Preparation (Hellebore-AgNPs System)

To prepare the hellebore-AgNPs system, the dried plant sample and AgNPs solution were mixed at a 1:2.5 mass ratio. Then, they were shaken (500 rpm) for 24 h at room temperature (22 °C). Subsequently, the obtained solution was filtered (0.45 μm) and dried in an oven at 30 °C for 4 h. Each experiment was repeated three times.

#### 3.3.2. Chitosan Phyto-Nanocarrier Preparation (Encapsulation Procedure)

The chitosan solution was prepared by dissolving 1.4 g of chitosan into 100 mL acetic acid solution (5% (*v*/*v*)). The resulting mixture was incubated at 80 °C for 48 h, under continuous stirring.

*Chitosan nanoparticles* (CN) (used to evaluated the encapsulation process)

The chitosan solution, cooled to room temperature, was added to 25 mL (2 M) NaOH solution dropwise with a Pasteur pipette, filtered and washed with deionized water.

To prepare *the first nanocarrier* (HC), a 0.25 g dried hellebore sample was added to 18 mL chitosan solution under continuous stirring at room temperature (22 °C). The mixture was added under stirring to 25 mL (2 M) NaOH solution dropwise with a Pasteur pipette, filtered and washed with deionized water.

*The hellebore-AgNPs nanocarrier* (HAgC) was prepared from 0.25 g hellebore-AgNPs and 18 mL chitosan solution, following an identical experimental procedure. The experiment was prepared in triplicate.

### 3.4. Characterization of Nanocarriers

#### 3.4.1. Fourier Transform Infrared (FT-IR) Spectroscopy

The data collection was performed after 20 recordings at a resolution of 4 cm^−1^ in the range of 4000–400 cm^−1^ on a Shimadzu AIM-9000 with ATR devices.

#### 3.4.2. XRD Spectroscopy

The X-ray powder diffraction (XRD) was performed using a Bruker AXS D8-Advance X-ray diffractometer (Bruker AXS GmbH, Karlsruhe, Germany) equipped with a rotating sample stage, Anton Paar TTK low-temperature cell (−180 °C ÷ 450 °C), high-vacuum, inert atmosphere, relative humidity control, and an Anton Paar TTK high-temperature cell (up to 1600 °C). The XRD patterns were compared with those from the ICDD Powder Diffraction Database (ICDD file 04-015-9120). The average crystallite size and the phase content were calculated using the whole-pattern profile-fitting method (WPPF).

#### 3.4.3. Scanning Electron Microscopy (SEM)

The SEM micrographs were obtained with an SEM-EDS system (JEOL JSM-IT200 Field Emission, Nieuw-Vennep, The Netherlands) equipped with a high-resolution electron gun and energy-dispersive X-ray spectrometer (EDS) with a MnK resolution of 133 eV.

#### 3.4.4. Dynamic Light Scattering (DLS) Particle Size Distribution Analysis

The DLS analysis was carried on a Microtrac/Nanotrac 252 (Montgomeryville, PA, USA), at room temperature (22 °C) and a scattering angle of 172°. Each experiment was repeated three times.

#### 3.4.5. Thermal Analysis

The thermal stability of the samples was investigated using a Thermal Analyzer produced by METTLER TOLEDO, model TGA/DSC3^+^ STARe System. The analyses were performed between 25 and 400 °C in a dynamic air atmosphere (20 mL/min, synthetic air) with a 10 °/min heating rate. Small amounts of every sample were placed in 40 μL aluminum melting crucibles. The DSC analysis was performed using a DSC 3+ Mettler Toledo in an air atmosphere (50 mL/min) in same range (25–400 °C).

#### 3.4.6. Antioxidant Activity

The antioxidant activity of the new nanocarriers (HC and HagC) and their components were evaluated using four different assays: 2,2-diphenyl-1-picrylhydrazyl (DPPH) radical scavenging assay; Folin–Ciocalteu assay; phosphomolybdate assay (total antioxidant capacity); and iron(III)-phenanthroline antioxidant assay. The experiments were carried out in 100 mL conical flasks containing 0.4 (±0.001) g dried sample to which 10 mL ethanol (70%) was added. The flasks were stirred (200 rpm), subjected to an ultrasonic extraction (45 min) and centrifuged (3500× *g* rpm, 10 min). Subsequently, the supernatant was collected and kept at −18 °C until further use in the selected antioxidant assays for this study.

### 3.5. Total Polyphenol Content Using the Folin–Ciocalteu Method

The total phenolic content in the new nanocarrier(s) and its components were determined spectrophotometrically according to the Folin–Ciocalteu procedure adapted from the literature [179]. A volume of 1.5 mL Folin–Ciocalteu reagent (2 M) and 0.5 mL Na_2_CO_3_ (2%) were added, dropwise with a Pasteur pipette, under stirring to 0.5 mL of each alcohol sample. The resulting solutions were kept at room temperature in a dark place for 60 min. All solutions were prepared in triplicate. The absorbance was measured at 725 nm using a UV-Vis spectrometer (DLAB SP-UV1000, Penjuru, Singapore). The phenol content was expressed in gallic acid equivalents (mg GAE/g sample) using a propyl gallate standard calibration curve between 9.375 µg/mL and 300 µg/mL in ethanol. The sample extract concentrations were calculated based on the linear equation obtained from the standard curve (y = 0.0015x + 0.2145 and the correlation coefficient (R^2^ = 0.9904).

### 3.6. DPPH Radical Scavenging Assay

The antioxidant activities of the new nanocarrier(s) and its components were comparatively evaluated using a DPPH (2,2-diphenyl-1-picrylhydrazyl) free radical elimination assay. A stock solution of 0.25 mM DPPH in ethanol was prepared. The sample extracts had dilutions between 20 µg/mL and 0.625 mg/mL. The ratio (*v*/*v*) of the DPPH to the samples was 3:1. The employed experimental procedure was the following: a volume of 1.5 mL 2,2-diphenyl-1-picrylhydrazyl reagent (2 M) was added, dropwise with a Pasteur pipette, under stirring to 0.5 mL of each alcohol sample. The resulting solutions were kept at room temperature (22 °C) for 30 min. The absorbance was measured at 515 nm using a UV-Vis spectrometer (DLAB SP-UV1000). All solutions were prepared in triplicate in ethanol, and 1:1 DPPH 0.1 mg/mL and ethanol was used as a control sample. The obtained results were used to calculate the average and the inhibition percentage (Inh%) (Equation (1)) [180].

Inh% = (A_0_ − A_l_)/A_0_ × 10 (1)

where:

- A_0_ = vehicle control absorbance;
- A_l_ = sample absorbance.

Further, the IC_50_ value was obtained from the inhibition percentage using the equation from the calibration curve generated for each sample and standard. The results are presented as the Inh% versus the concentration (mg/mL) [181].

### 3.7. Phosphomolybdate Assay (Total Antioxidant Capacity)

The total antioxidant capacity assay of the new nanocarrier(s) and its components was carried out with the phosphomolybdenum procedure using ascorbic acid as the standard [182]. A volume of 6 mL reagent solution (0.6 M sulfuric acid, 28 mM sodium phosphate and 4 mM ammonium molybdate) and 0.6 mL of each sample were placed into a water bath at 90 °C for 120 min. Next, the mixed solutions were cooled at room temperature (22 °C). The absorbance was measured at 765 nm using a UV-Vis Perkin-Elmer Lambda 35 (Perkin Elmer, Waltham, MA, USA).

A blank solution was used in which 6 mL of reagent was added to 0.6 mL of methanol, and then the mixture was incubated under the same experimental conditions: 90 °C for 120 min and then cooled at room temperature (22 °C). The total antioxidant capacity was determined according to the following equation (Equation (2)):

Total antioxidant capacity (%) = (Abs. of control − Abs. of sample)/(Abs. of control) × 100(2)

The results are presented as μg/mL of ascorbic acid equivalents (AAE).

### 3.8. Iron(III)-Phenanthroline Antioxidant Assay (OPM)

The ferric ion reduction capacity of the new nanocarrier(s) and its components were comparatively evaluated according to the following procedure: in 25 mL mL Erlenmeyer flasks containing 1 mL 1,10-phenanthroline (0.1%), 1 mL FeCl_3_•6H_2_O (0.1%) and 7 mL distilled water, 1 mL of each sample was added. The resulting solution was stirred at room temperature (22 °C) for 25 min. The absorbance was measured at 510 nm using a UV-Vis spectrometer (DLAB SP-UV1000, DLAB Scientific Inc., Reverside, CA, USA). The ferric ion reduction capacity of the extracts is expressed in mmol Fe/g dry product using a FeSO_4_•7H_2_O calibration curve between 0 and 500 µmol/L in distilled water [183].

The results are reported according to the concentrations on the standard curve (y = 0.0015x + 0.1795, (correlation coefficient) R^2^ = 0.9727.

### 3.9. Encapsulation Yield, Encapsulation Efficiency and Loading Capacity

The *encapsulation yield* (*EY*%) was determined as the ratio of the weight of the material nanoencapsulated into chitosan and the raw materials’ weight, according to the following equation (Equation (3)) [174]:

(3) $$EY\;\left(\%\right)=\frac{weight\;of\;nanocarrier\;(g)}{weigh\;raw\;materials\;(g)}\;\times \;100$$

### 3.10. Encapsulation Efficiency (EE%)

The encapsulation efficiency (*EE*%) was calculated as the percent of the total amount (*g*) of the hellebore and hellebore-AgNPs system based on the total amount (*g*) used as raw materials in the encapsulation process (Equation (4)) [184,185].

(4) $$EE\;\left(\%\right)=\frac{amount\;of\;compound\;encapsulated\;(g)}{amount\;compound\;used\;as\;raw\;materials\;(g)}\times 100\;$$

The compound contents and encapsulation efficiency (*EE*%) of the chitosan nanoparticles were determined using a UV-Vis Perkin-Elmer Lambda 35 (Perkin Elmer, Waltham, MA, USA). All absorbance measurements were conducted with a 10 mm UV/Vis spectroscopy cell at room temperature, using a solvent (ethanol/chloroform = 1:1) as a blank. The employed experimental procedure was the following: The samples (15 mg) were subject to sonication extraction (frequency of 50 kHz) in 20 mL of solvent (HCl/ethanol/chloroform = 3:2:2) for 40 min at room temperature (22 °C) and then centrifuged. Subsequently, the supernatant’s concentration was determined using atomic UV-Vis spectroscopy. Each experiment was repeated three times.

### 3.11. Preparation of the Curves of the Concentrations of the Loaded Materials (Hellebore and Hellebore-AgNPs System)

The ultrasonic-assisted extraction experiments were carried out with five different amounts of each sample (0.01–0.20 mg) using a constant volume of solvent (ethanol/chloroform = 1:1) for 40 min at 22 °C, followed by centrifugation. Subsequently, the concentrations of the loaded materials in the filtrate were determined with UV-Vis spectroscopy. Calibration curves were plotted for hellebore and the hellebore-AgNPs system. The correlation coefficients, which were R^2^ = 0.9896 (hellebore) and R^2^ = 0.9932 (hellebore-AgNPs system), demonstrate the good linear relationship of the data. Each experiment was repeated three times. The amount of each compound encapsulated in the chitosan nanoparticles was obtained from the standard curve of the concentration versus its absorbency.

*The encapsulated content* (*EC%*) was calculated according to the following equation (Equation (5)) [184,185]:

(5) $$EC\;\left(\%\right)=\frac{concentration\;compound\;encapsulated\;x\;volume\;solution\;}{amount\;compound\;used\;as\;raw\;materials\;\left(g\right)}\times 100\;$$

The concentration of each compound was determined using UV-Vis spectroscopy according to the above experimental procedure.

### 3.12. Statistical Analysis

Each experimental set was performed in triplicate, using one-way analysis of variance (ANOVA) without replication with Scheffe’s post hoc test comparison; *p* < 0.05 was taken as statistically significant.

## 4. Conclusions

The undertaken study describes the low-molecular-mass metabolite profiling of *Helleborus purpurascens* growing wild in Romania. The biological activities of each category of phytoconstituents were discussed. Furthermore, a new phyto-carrier system (hellebore-AgNPs system) with unique physical–chemical characteristics and high antioxidant activity was prepared. The encapsulation potential of the plant samples (i.e., hellebore) and new phyto-carrier system (hellebore-AgNPs system) in chitosan nanoparticles (CNs) was evaluated. The development of two new phyto-nanocarriers, HC and HAgC, was confirmed through FTIR, EDX, XRD, DLS and SEM studies. The thermal stability was investigated. Several different antioxidant assays (DPPH, Folin–Ciocalteu, phosphomolybdate (total antioxidant capacity) and iron(III)-phenanthroline antioxidant) were carried out to ensure that HC and HAgC exhibited potent antioxidant activity. Collectively, this study shows the potential advantages of these phyto-nanocarriers encapsulated as selective therapeutic delivery systems. Nonetheless, further studies are necessary to investigate the in vitro release, biological properties and bioavailability of these new phyto-nanocarriers.

## Figures and Tables

**Table 1:** Phytochemicals identified via GC–MS analysis of the hellebore sample.

No.	Retention Time (RT) (min)	Retention Index (RI) Determined	Adams Index (AI)	Area (%)	Compound Name	Ref.
**1**	11.43	1972	1983	2.18	palmitic acid	[47,48,49]
**2**	11.98	1598	1602	5.32	hexadecane	[47]
**3**	14.61	3160	3168	6.76	stigmasterol	[47,50]
**4**	15.87	3195	3213	9.85	sitosterol	[47,50]
**5**	17.22	2543	2634	8.53	stearic acid	[47,48,49]
**6**	25.96	2078	2123	21.84	phytol	[47,50]
**7**	26.88	2848	2861	35.69	squalene	[47,50]
**8**	28.45	2688	2694	6.58	heptacosane	[47,48]

RI—retention index calculated based on a calibration curve of a C8–C20 alkane standard mixture.

**Table 2:** The secondary metabolites identified in the mass spectrometry analysis in hellebore sample.

No.	*m*/*z* Detected	Theoretic *m*/*z*	Formula	Tentative of Identification	Category	Ref.
1	76.07	75.07	C_2_H_5_NO_2_	glycine	amino acids	[3,4,11]
2	89.88	89.09	C_3_H_7_NO_2_	alanine	amino acids	[4,11,13]
3	96.09	96.08	C_5_H_4_O_2_	protoanemonin	lactones	[2,6,11]
4	116.15	116.16	C_6_H_12_O_2_	caproic acid	fatty acids	[47]
5	119.11	119.12	C_4_H_9_NO_3_	threonine	amino acids	[3,4,11]
6	131.17	131.17	C_6_H_13_NO_2_	isoleucine	amino acids	[3,4,11]
7	132.15	132.16	C_5_H_12_N_2_O_2_	ornithine	amino acids	[3,4,11]
8	147.13	146.14	C_5_H_10_N_2_O_3_	alanylglycine	peptides	[4,11]
9	148.12	147.13	C_5_H_9_NO_4_	glutamic acid	amino acids	[3,4,11]
10	151.14	150.13	C_5_H_10_O_5_	arabinose	carbohydrates	[51,52]
11	156.16	155.15	C_6_H_9_N_3_O_2_	histidine	amino acids	[3,4,11]
12	155.15	164.16	C_9_H_8_O_3_	p-coumaric acid	phenolic acids	[3,10,53]
13	166.18	165.19	C_9_H_11_NO_2_	phenylalanine	amino acids	[3,4,11]
14	167.16	166.17	C_9_H_10_O_3_	3-phenyllactic acid	organic acid	[52,53]
15	171.11	170.12	C_7_H_6_O_5_	gallic acid	phenolic acids	[3,10,53]
16	175.19	174.20	C_6_H_14_N_4_O_2_	arginine	amino acids	[4,11]
17	177.18	176.17	C_6_H_12_N_2_O_4_	glycylthreonine	peptides	[4,11]
18	181.17	180.16	C_9_H_8_O_4_	caffeic acid	phenolic acids	[3,10,53]
19	193.16	192.17	C_10_H_8_O_4_	anemonin	butenolides	[2,3,51]
20	195.18	194.18	C_10_H_10_O_4_	ferulic acid	phenolic acids	[3,10,52]
21	201.33	200.32	C_12_H_24_O_2_	lauric acid	fatty acids	[47]
22	203.26	202.25	C_9_H_18_N_2_O_3_	alanyl-isoleucine	peptides	[4,11]
23	205.21	204.22	C_11_H_12_N_2_O_2_	tryptophan	amino acids	[3,4,11]
24	213.22	212.21	C_8_H_12_N_4_O_3_	glycyl-histidine	peptides	[4,11]
25	218.26	217.27	C_9_H_19_N_3_O_3_	alanyl-lysine	peptides	[4,11]
26	220.23	219.24	C_8_H_17_N_3_O_4_	ornithinoalanine	peptides	[4,11]
27	221.29	220.29	C_8_H_16_N_2_O_3_S	alanylmethionine	peptides	[4,11]
28	225.29	224.30	C_6_H_12_N_2_O_3_S_2_	cysteylcysteine	peptides	[4,11]
29	227.45	226.44	C_16_H_34_	hexadecane	hydrocarbons	[54]
30	229.23	228.24	C_14_H_12_O_3_	resveratrol	stilbenoids	[3,10]
31	241.29	240.30	C_6_H_12_N_2_O_4_S_2_	cystine	amino acids	[3,4,11]
32	244.31	243.30	C_11_H_21_N_3_O_3_	lysylproline	peptides	[4,11]
33	245.19	244.20	C_9_H_12_N_2_O_6_	uridine	nucleosides	[55]
34	249.35	248.34	C_10_H_20_N_2_O_3_S	valyl-methionine	peptides	[4,11]
35	253.39	252.40	C_8_H_16_N_2_O_3_S_2_	cysteylmethionine	peptides	[4,11]
36	255.28	254.29	C_11_H_18_N_4_O_3_	histidylvaline	peptides	[4,11]
37	257.24	256.25	C_12_H_16_O_6_	phenylglucoside	glycosides	[55]
38	257.43	256.42	C_16_H_32_O_2_	palmitic acid	fatty acids	[47]
39	260.35	259.35	C_12_H_25_N_3_O_3_	leucyllysine	peptides	[4,11]
40	262.27	261.28	C_13_H_15_N_3_O_3_	tryptophylglycine	peptides	[4,11]
41	269.33	268.33	C_12_H_16_N_2_O_3_S	phenylalanylcysteine	peptides	[4,11]
42	270.25	269.26	C_10_H_15_N_5_O_4_	asparaginyl-Histidine	peptides	[4,11]
43	271.25	270.24	C_15_H_10_O_5_	apigenin	flavonoids	[55]
44	271.49	270.50	C_17_H_34_O_2_	margaric acid	fatty acids	[47]
45	276.31	275.30	C_14_H_17_N_3_O_3_	tryptophylalanine	peptides	[4,11]
46	277.25	276.24	C_11_H_16_O_8_	ranunculin	glycosides	[51,56,57]
47	278.35	277.35	C_9_H_19_N_5_O_3_S	cysteinyl-arginine	peptides	[4,11]
48	279.34	278.35	C_15_H_22_N_2_O_3_	phenylalanylleucine	peptides	[4,11]
49	284.28	283.28	C_11_H_17_N_5_O_4_	alanyl-glycyl-histidine	peptides	[4,11]
50	284.49	284.50	C_18_H_36_O_2_	stearic acid	fatty acids	[46]
51	287.23	286.24	C_15_H_10_O_6_	kaempferol	flavonoids	[3,10,58,59]
52	287.34	286.35	C_11_H_18_N_4_O_3_S	histidylmethionine	peptides	[4,11]
53	288.37	287.36	C_12_H_25_N_5_O_3_	leucylarginine	peptides	[4,11]
54	291.26	290.27	C_15_H_14_O_6_	epicatechin	flavonoids	[3,10]
55	294.31	293.32	C_14_H_19_N_3_O_4_	phenylalanylglutamine	peptides	[4,11]
56	295.29	294.30	C_14_H_18_N_2_O_5_	glutamyl-phenylalanine	peptides	[4,11]
57	296.28	295.29	C_13_H_17_N_3_O_5_	tyrosylglycylglycine	peptides	[4,11]
58	297.49	296.50	C_20_H_40_O	phytol	terpenoids	[57]
59	303.24	302.23	C_15_H_10_O_7_	quercetin	flavonoids	[3,59]
60	306.39	305.40	C_12_H_23_N_3_O_4_S	arginylmethionine	peptides	[4,11]
61	308.36	307.37	C_11_H_21_N_3_O_5_S	glycyl-threonyl-methionine	peptides	[4,11]
62	309.51	308.50	C_20_H_36_O_2_	eicosadienoic acid	fatty acids	[54]
63	312.33	311.34	C_12_H_21_N_7_O_3_	histidinyl-arginine	peptides	[4,11]
64	320.41	319.42	C_13_H_25_N_3_O_4_S	leucyl-methionyl-glycine	peptides	[4,11]
65	323.36	321.37	C_15_H_23_N_5_O_3_	arginylphenylalanine	peptides	[4,11]
66	333.34	332.35	C_16_H_20_N_4_O_4_	glycyl-tryptophanyl-alanine	peptides	[4,11]
67	335.41	334.40	C_11_H_22_N_6_O_4_S	glycyl-arginyl-cysteine	peptides	[4,11]
68	338.37	337.37	C_16_H_23_N_3_O_5_	threonyl-phenylalanyl-alanine	peptides	[4,11]
69	341.59	340.60	C_22_H_44_O_2_	behenic acid	fatty acids	[47]
70	343.39	342.40	C_20_H_24_NO_4_^+^	magnoflorine	alkaloids	[51]
71	349.41	348.42	C_12_H_24_N_6_O_4_S	arginyl-alanyl-cysteine	peptides	[4,11]
72	355.49	354.50	C_24_H_34_O_2_	bufadienolide	terpenoids	[60]
73	357.34	356.33	C_13_H_20_N_6_O_6_	asparaginyl-seryl-histidine	peptides	[4,11]
74	361.29	360.30	C_18_H_16_O_8_	rosmarinic acid	phenolic acids	[10]
75	365.41	364.40	C_18_H_28_N_4_O_4_	lysyl-phenylalanyl-alanine	peptides	[4,11]
76	368.51	367.50	C_17_H_25_N_3_O_4_S	alanyl-methionyl-phenylalanine	peptides	[4,11]
77	370.41	369.40	C_16_H_23_N_3_O_5_S	methionyl-tyrosyl-glycine	peptides	[4,11]
78	374.41	373.40	C_18_H_23_N_5_O_4_	phenylalanyl-histidyl-alanine	peptides	[4,11]
79	381.69	380.70	C_27_H_56_	heptacosane	hydrocarbons	[47]
80	383.43	382.42	C_16_H_26_N_6_O_5_	leucyl-asparaginyl-histidine	peptides	[4,11]
81	384.37	383.36	C_14_H_21_N_7_O_6_	histidyl-asparaginyl-asparagine	peptides	[4,11]
82	367.71	386.70	C_27_H_46_O	furostan	terpenoids	[2,3]
83	393.51	392.50	C_18_H_24_N_4_O_4_S	tryptophanyl-methionyl-glycine	peptides	[4,11]
84	398.49	397.50	C_18_H_27_N_3_O_5_S	threonyl-phenylalanyl-methionine	peptides	[4,11]
85	401.59	400.60	C_27_H_44_O_2_	spirostan	saponins	[2,3]
86	406.51	405.50	C_15_H_31_N_7_O_4_S	lysyl-arginyl-cysteine	peptides	[4,11]
87	408.50	407.50	C_20_H_29_N_3_O_6_	glutamyl-isoleucyl-phenylalanine	peptides	[4,11]
88	411.69	410.70	C_30_H_50_	squalene	terpenoids	[47]
89	413.70	412.70	C_29_H_48_O	stigmasterol	sterols	[47]
90	415.69	414.70	C_29_H_50_O	sitosterol	sterols	[47]
91	417.51	416.50	C_24_H_32_O_6_	hellebrigenin	terpenoids	[2,3,61]
92	434.49	433.50	C_15_H_31_N_9_O_4_S	arginyl-arginyl-cysteine	peptides	[4,11]
93	450.51	449.50	C_15_H_27_N_7_O_7_S	arginyl-glycyl-aspartyl-cysteine	peptides	[4,11]
94	453.49	452.50	C_21_H_36_N_6_O_5_	alanyl-leucyl-leucyl-histidine	peptides	[4,11]
95	463.61	462.60	C_27_H_42_O_6_	shidasterone	phytoecdysteroids	[62,63]
96	465.39	464.40	C_21_H_20_O_12_	hyperoside	flavonoids	[9]
97	469.51	468.50	C_24_H_32_N_6_O_4_	phenylalanyl-arginyl-phenylalanine	peptides	[4,11]
98	481.59	480.60	C_27_H_44_O_7_	ecdysterone	terpenoids	[2,3,9,64]
99	482.61	481.60	C_16_H_31_N_7_O_6_S_2_	arginyl-threonyl-cysteinyl-cysteine	peptides	[4,11]
100	494.51	493.50	C_21_H_31_N_7_O_7_	arginyl-glycyl-aspartyl-phenylalanine	peptides	[4,11]
101	521.59	520.60	C_24_H_40_N_8_O_5_	H-Lys-Ala-Phe-Arg-OH	peptides	[4,11]
102	563.59	562.60	C_30_H_42_O_10_	degluco-hellebrin	terpenoids	[60,64]
103	572.69	571.70	C_27_H_37_N_7_O_5_S	arginyl-phenylalanyl-phenylalanyl-cysteine	peptides	[4,11]
104	611.49	610.50	C_27_H_30_O_16_	rutin	flavonoids	[3,9]
105	725.81	724.80	C_36_H_52_O_15_	hellebrin	terpenoids	[2,3]

**Table 3:** The characteristic absorption bands attributed to the secondary metabolites identified in *Helleborus purpurasces*.

Secondary Metabolites	Wavenumber (cm^−1^)	Ref.
bufadienolides	3560, 3035, 1740–1760, 1718–1736	[110,111]
butenolide	1770, 1605, 830	[83,112]
phytoecdysteroids	3393, 3446, 1652	[113,114]
peptides	3300, 3100, 1695–1610, 1575–1480,1320–1220, 800–640, 765–625, 605–535	[115,116]
terpenoids	2939, 1740, 1651, 810	[117]
fatty acids	3020–3010, 2924–2915, 2855–2847, 2800–2900, 1746, 1710, 1250, 720	[118,119]
flavonoids	4000–3125, 3140–3000, 1670–1620, 1650–1600, 1600–1500, 1450–1490	[120,121]
phenolic acids	1800–1650, 1734, 1720, 1627, 1522, 1440, 1410, 1420–1300, 1367, 1315, 1255, 1170–1100	[117]
phytosterols	3427, 2937, 1466, 1383, 1192, 1063, 740.5	[122]
carbohydrates	3524, 3324, 2943, 2917, 2874, 1129, 1089, 1048, 1016, 991, 782, 671, 602	[123]
amino acids	3400; 3330–3130; 2530–2760; 2130; 1724–1754, 1687, 1675, 1663, 1652, 1644, 1632, 1621, 1611, 1500–1600	[124]
alkaloids	3362, 1645.9, 1515.7, 1283, 1250.5	[125]
nucleoside	3351, 3104, 2925, 2800, 1670, 1470, 1396, 1269, 1209, 1137. 1095, 1053, 980, 906, 830, 766, 572, 451	[126]
glycosides	3401, 1711, 1450, 1073, 991, 914, 863.5; 769, 743, 684, 630, 432	[127]
stilbenoids	1605, 1583, 1380, 960	[128,129]

**Table 4:** The antioxidant assay results for hellebore, chitosan, hellebore-AgNPs system and HC and HAgC phyto-nanocarriers.

Sample Name	IC_50_ (mg/mL)	Total Phenolic Content (mg GAE/mL)	mg AAE/mL	mmol Fe/g
hellebore	3.86 ± 0.16	5.16 ± 0.18	91.41 ± 0.012	34.91 ± 0.024
chitosan	17.21± 0.09	1.82 ± −1.24	15.16 ± 0.014	9.16 ± 0.021
HC phyto-nanocarrier	4.17 ± 0.12	4.85 ± 0.14	100.48 ± 0.017	27.75 ± 0.013
hellebore-AgNPs system	2.35 ± 0.15	9.41 ± 0.17	156.87 ± 0.019	48.08 ± 0.027
HAgC phyto-nanocarrier	2.56 ± 0.14	8.93 ± 0.15	186.98 ± 0.021	41.34 ± 0.018

**Table 5:** Parameters of the encapsulation process for the newly prepared phyto-nanocarriers.

Sample Name	*EE* (%)	*EC* (%)	*EY* (%)
CN	-	-	70.34 ± 0.23
HC	82.45 ± 0. 21	81.65 ± 0.35	87.45 ± 0.35
HAgC	91.05 ± 0.18	92.18 ± 0.28	95.34 ± 0.48

## Data Availability

All data are contained within the article.

## Supplementary Materials

The following supporting information can be downloaded at: https://www.mdpi.com/article/10.3390/plants12193479/s1. Figure 1S: TIC chromatogram of hellebore extract. Figure 2S: The mass spectrum of Helleborus purpurascens sample.
