# Supplementary material for: Wild-Grown Romanian Helleborus purpurascens Approach to Novel Chitosan Phyto-Nanocarriers—Metabolite Profile and Antioxidant Properties
Source: Plants (Basel). 2023 Oct 4;12(19):3479. doi: 10.3390/plants12193479 (PMC10574898; doi:10.3390/plants12193479)
Supplement: Supplementary file 1 [file plants-12-03479-s001.zip › plants-2632406-supplementary.pdf]

SUPPLEMENTARY INFORMATION

Wild-grown Romanian *Helleborus purpurascens* approach to novel chitosan phyto-nanocarriers – metabolite profile and antioxidant properties

Adina-Elena SEGNEANU[1*], Gabriela VLASE[1,2], Titus VLASE[1,2], Crina Andreea SICOE[3], Maria Viorica CIOCALTEU[4], Daniel Dumitru HEREA[5], Ovidiu-Florin GHIRLEA[7], Ioan GROZESCU[6] and Valentin NANESCU[4]

FIGURES

Figure 1S: TIC chromatogram of hellebore extract

Figure 2S: The mass spectrum of *Helleborus purpurascens* sample

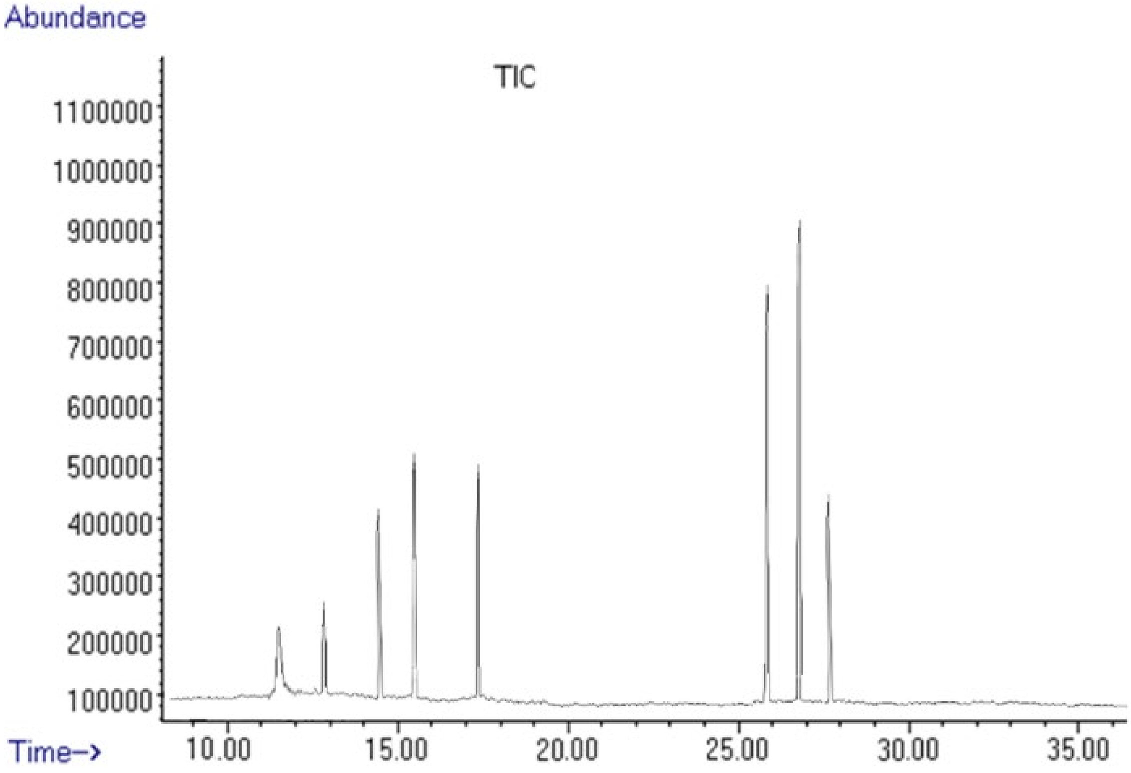

Figure 1S

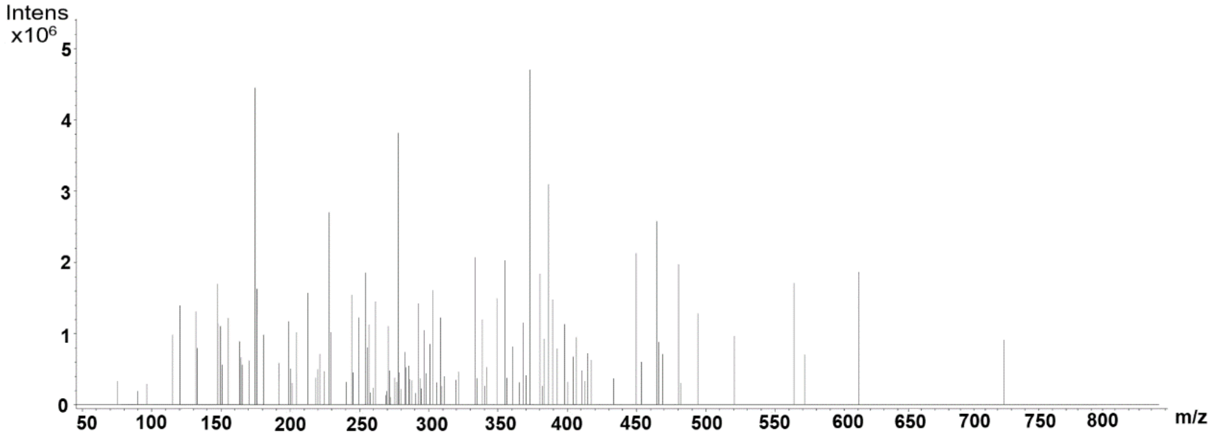
Intens
x10^6
5
4
3
2
1
0
50
100
150
200
250
300
350
400
450
500
550
600
650
700
750
800
m/z

Figure 2S
